# The role of resistance exercise-induced local metabolic stress in mediating systemic health and functional adaptations: could condensed training volume unlock greater benefits beyond time efficiency?

**DOI:** 10.3389/fphys.2025.1549609

**Published:** 2025-04-17

**Authors:** Ivan Curovic

**Affiliations:** Institute of Coaching and Performance, University of Central Lancashire, Preston, United Kingdom

**Keywords:** hypertrophy, strength, muscular endurance, superset, blood flow restriction, anaerobic, lactate

## Abstract

The majority of “specialised” exercise configurations (e.g., supersets, drop sets, blood flow restriction) are being assessed as “shortcuts” to hypertrophy and strength improvements. However, these advanced training techniques may also offer significant benefits for systemic health and functional outcomes across recreational and clinical populations via locally induced metabolic responses. Stress-regulating mechanisms are known to enhance the body’s resilience by facilitating allostasis, the process of coordinating adaptive processes in reaction to stressors such as physical training. Yet, the role of the local metabolic stress provoked by resistance exercise has not gained much research attention despite its wide potential. Positive effects are not only linked to improved muscular endurance, hypertrophy and strength via primary and secondary mechanisms, but also to the release of myokines, hormones, microRNAs, immune factors, inflammatory substances and other endocrine molecules that initiate numerous health-promoting modifications on a systemic level. Resistance exercise strategies that maximise the local accumulation of metabolites are not well defined, although high volume, close proximity to failure and shorter rests seem to be a necessity. Additionally, blood flow restriction training provides a potent alternative for inducing local acidosis, thereby triggering several pathways associated with improved immunity and physical function even in remote muscle tissues. Future research is warranted to further explore advanced resistance training techniques, as these approaches may offer comparable benefits for physical and mental health to those seen with other forms of exercise such as high-intensity interval training and heavy resistance training.

## Highlights

•Resistance exercise-induced local metabolic stress facilitates improvements in strength, hypertrophy and muscular endurance with the light-to-moderate loads. Some of these adaptations also occur in muscles trained for strength separately within the same session.
•Resistance exercise-induced local metabolic stress stimulates a comprehensive release of various myokines and cytokines associated with multiple health benefits on a systemic level. These benefits include blood pressure reductions, metabolic health and potential enhancement of cognitive function.
•The evidenced benefits are realised directly (via metabolites) and indirectly (via mechanical tension). In both cases, local metabolic stress is the key factor of influence in the described protocols.
•Local metabolic stress appears to be maximised by high volume of fatiguing sets. Blood flow restriction exercise augments anaerobic conditions, thereby allowing for a very light resistance to be applied.

## 1 Introduction

Physical exercise of any type is associated with improved health and life expectancy (Pedersen, 2019; Carapeto and Aguayo-Mazzucato, 2021; Kraschnewski et al., 2016). While moderate aerobic activities demonstrate the highest dose-dependent effect (O’Keefe et al., 2023), vigorous forms of exercise such as high-intensity interval training (HIIT) offer augmented health and functional benefits for the organism through enhanced aerobic and anaerobic adaptations (Atakan et al., 2021). High frequency of intense whole-body endurance exercise forms, however, may not be the best option for life-extending practices among sedentary and older individuals (O’Keefe et al., 2023; Franklin et al., 2020) due to the potential cardiac overuse injury risk (Franklin et al., 2020). On the other hand, regular resistance training has been increasingly associated with improved longevity (Kraschnewski et al., 2016; Shailendra et al., 2022; Momma et al., 2022) and physical function (Pedersen, 2019; Valenzuela et al., 2023) as well as with reduced risk of metabolic and musculoskeletal diseases (Kraschnewski et al., 2016; Shailendra et al., 2022; Momma et al., 2022; Westcott, 2012). While exercise strategies targeting maximal strength capacity dominate research, low-load training’s significance is comparatively underappreciated (Weakley et al., 2023). Yet, training with lighter resistances performed to fatigue could offer significant benefits for recreational population, including those at risk of chronic diseases (Weakley et al., 2023; Yang et al., 2019). These benefits stem from numerous adaptations commonly associated with both traditional strength training and aerobic endurance exercise (Weakley et al., 2023).

Metabolic stress by exercise is characterised by the accumulation of metabolites like lactate, inorganic phosphate and hydrogen ions within muscle cells, resulting from the low energy availability caused by high-intensity demands on energy systems (de Freitas et al., 2017). It can be systemically provoked with strenuous forms of whole-body movements (Atakan et al., 2021), or locally when there is a sustained muscular contraction or restricted blood flow in the targeted muscles (Lawson et al., 2022). While the former exercise modality places significant demands on the cardiovascular system (O’Keefe et al., 2023), the latter focuses on specific muscle tissues thereby maximising the anaerobic conditions regionally (Lawson et al., 2022). This local metabolic stress (LMS) is associated with the augmented production of lactate (Robergs et al., 2004), reactive oxygen species (ROS) (de Freitas et al., 2017) and serum hormones (Goto et al., 2005), contributing to the generation of myokines (Laurens et al., 2020), exerkines (Saheli et al., 2024) and microRNA (Domańska-Senderowska et al., 2019). All these molecules are released into systemic circulation by the exercising muscles and organs, leading to multiple health benefits (Saheli et al., 2024; So et al., 2014; Brooks et al., 2023). Unfortunately, muscular endurance training type, which embodies the principles of LMS (Goto et al., 2005), has primarily been investigated in relation to the protocols examining strength and hypertrophy outcomes (Hickmott et al., 2022; Haugen et al., 2023; Lacio et al., 2021; Warneke et al., 2024; Sødal et al., 2023; Ozaki et al., 2016), while the associations to health and longevity been rarely distinguished between different resistance training modalities (Kraschnewski et al., 2016; Shailendra et al., 2022; Momma et al., 2022).

Local anaerobic stimulus in exercised muscles is likely to be behind the reported significant improvements in blood pressure from isometric and blood flow resistance (BFR) training in recreational populations (Devereux, 2010; Feng et al., 2024). Furthermore, the capacity for local fatigue resistance serves as a practical measure of neuromuscular function, offering valuable insights into the ability to sustain normal activities, particularly in older adults (Devereux, 2010; Bemben, 1998; Kahraman and Varol, 2024; Bemben et al., 1996). Interestingly, both moderate-load exercise and light-load exercise with and without BFR lead to hypertrophy comparable with traditional strength training only if exercises are performed to near the failure points (Weakley et al., 2023; Lixandrão et al., 2018; Keskin et al., 2025; Schoenfeld et al., 2017; Grgic et al., 2018; Robinson et al., 2024), displaying less reliable results for strength increases (Weakley et al., 2023; Lixandrão et al., 2018; Schoenfeld et al., 2017). A recent systematic review and meta-analysis suggested that LMS might be behind the strength, hypertrophy, and endurance improvements observed with superset protocols as evidenced by higher blood lactate levels and energy expenditure (Zhang et al., 2025). General resistance training has demonstrated positive influence on oxidative stress and the liver (Bucarey et al., 2024), inflammation (Kwon et al., 2020; Brandt and Pedersen, 2010), immune system (Kwon et al., 2020; Chow et al., 2022; Zunner et al., 2022), cognition (Saheli et al., 2024; Bu et al., 2025; Huang et al., 2021), metabolic health (Bucarey et al., 2024; Kwon et al., 2020; Chow et al., 2022), cardiovascular system (Devereux, 2010; Chow et al., 2022; Wang et al., 2018; Owen et al., 2010), and has exerted a reduced risk of infections and chronic diseases, including diabetes, cancer, atherosclerosis and neurodegeneration (Brandt and Pedersen, 2010; Zunner et al., 2022; Saliminejad et al., 2019). Whether local metabolic responses in isolated muscle groups contribute to these outcomes remains underexamined, but plausible (Weakley et al., 2023; Goto et al., 2005; Devereux, 2010; Zhang et al., 2025; May et al., 2018; Farrell et al., 2018; Coffey and Hawley, 2007; Spiering et al., 2009; Kraemer and Ratamess, 2005; Fiorenza et al., 2018; Liu et al., 2024). This is particularly highlighted by studies with BFR exercise protocols, which show a remarkable potential to influence a plethora of positive adaptations on a whole-body level instigated by induced local acidosis (Feng et al., 2024; Lixandrão et al., 2018; Martin et al., 2022; Saatmann et al., 2021; Hedt et al., 2022). For example, there is evidence that exercise-provoked LMS could facilitate morphological and functional adaptations in remote muscle tissues (Spiering et al., 2009; Curovic et al., 2024; Cook et al., 2014; Rønnestad et al., 2011; Spillane et al., 2015), and offer a potential for therapeutic influence, significantly reducing pain and functional disability in athletes with chronic non-specific low back pain (Liu et al., 2024).

Exercise focused on specific muscle groups in close proximity to each other produce hypoxia-induced LMS without overburdening the cardiovascular system (Goto et al., 2005; Feng et al., 2024; Martin et al., 2022), potentially making it accessible to recreational individuals. Moreover, unlike athletes who aim to optimise maximal strength and power gains (Van Hooren et al., 2024), recreational people and older adults may benefit from lighter loads to minimise the risk of injury with the reduced strain on connective tissues (Weakley et al., 2023; Bemben, 1998). While low-load training to failure may cause more discomfort than training with heavy loads (Fisher and Steele, 2017), stopping just short of failure may be better tolerated with light loads (de Lemos Muller et al., 2019), and might be more sustainable compared to HIIT protocols (Roy et al., 2018). Therefore, the purpose of this narrative review is to explore the role of resistance exercise-induced LMS in promoting systemic health and functional adaptations and evaluate the usefulness of resistance training modalities to maximise local-to-systemic endocrine responses.

## 2 Resistance exercise, metabolic stress and secretory factors

As mentioned above, LMS arises when oxygen availability is insufficient in the exercised muscles, leading to a greater reliance on glycolysis for ATP production (de Freitas et al., 2017; Hwang and Willoughby, 2019). In this scenario, sustained contractions create a localised shift from aerobic to anaerobic conditions, resulting in the accumulation of metabolites that exert influence on both local and distant muscle cells, neurons, and organs (Brooks et al., 2023; Huang et al., 2021; Rønnestad et al., 2011; Godfrey et al., 2009; Madarame et al., 2008). Therefore, to elicit a metabolic response in targeted muscles, resistance exercise must induce anaerobic conditions (de Freitas et al., 2017; Lawson et al., 2022; Robergs et al., 2004; Goto et al., 2005). However, heavy loads do not place significant demands on the glycolytic system because they primarily rely on phosphocreatine for maximal, short-duration contractions (de Poli et al., 2019; Hargreaves and Spriet, 2020; Krisanda et al., 1988). This is due to the faster onset of volitional failure caused by the inability to sustain high levels of type 2 fibre recruitment necessary for intense contractions (Krisanda et al., 1988; Hultman and Greenhaff, 1991). In contrast, when a muscle is actively performing against lighter loads, the primary physiological mechanisms for energy requirements arise from oxidative and glycolytic pathways (Lawson et al., 2022; Krisanda et al., 1988). Indeed, according to Henneman’s size principle, motor units are activated in a sequential order starting from smaller, oxygen-dependent type 1 fibres, followed by the larger glycolytic type 2 fibres that are progressively engaged to compensate for the declining force output with fatigue advancement (Wernbom and Aagaard, 2020). Alternatively, when exercise is performed with high velocity against light resistance, the central nervous system can prime the muscle with maximal intent to achieve the greatest possible contraction speed, overriding the typical size principle recruitment sequence (Sale, 1988; Maffiuletti et al., 2016). This process involves a high neural discharge rate, which allows for the rapid activation of type 2 fibres (Maffiuletti et al., 2016; Aagaard et al., 2002), where velocity is prioritised over force and contractions slow down relatively quickly (Boccia et al., 2024). Despite the differences in these recruitment dynamics, the end result of using light resistance remains similar if repetitions are performed until near the failure point – elevated LMS facilitating a complete muscle fibre activation (Lawson et al., 2022; Morton et al., 2019).

In simplified physiological terms, as exercise duration extends, anaerobic glycolysis becomes increasingly engaged to meet energy demands, outpacing oxidative phosphorylation and phosphagen system (Krisanda et al., 1988; Koopman et al., 2006; Koh et al., 2022). This shift results in a transient mismatch between the rate of pyruvate production and its utilisation by aerobic mechanisms (Melkonian and Schury, 2024). During anaerobic glycolysis, pyruvate is converted into lactate which is then used to regenerate nicotinamide adenine dinucleotide (NAD) from its reduced form (NADH) to the oxidised form (NAD+), thereby allowing the energy production process to continue (Melkonian and Schury, 2024). This regeneration is vital for sustaining glycolysis during high-intensity activity (Melkonian and Schury, 2024). However, as muscle contractions persist, NADH levels increase, eventually overwhelming the capacity of lactate to remove hydrogen ions (Melkonian and Schury, 2024). Consequently, the disruption in the pH balance (i.e., muscle acidosis) becomes one of the main limiting factor of muscular contractions (Melkonian and Schury, 2024; Theofilidis et al., 2018). The local hypoxic conditions achieved initiate a whole cascade of events, including the release of various molecules and anabolic hormones (Goto et al., 2005; Hwang and Willoughby, 2019; Hjortshoej et al., 2023), intracellular signalling pathways (Lixandrão et al., 2018), and altered gene expression with satellite cell activity (Goto et al., 2005; Hwang and Willoughby, 2019; Hjortshoej et al., 2023). Notably, these pathways are not only facilitated by LMS in isolation, but also by the mechanical tension inflicted on type II fibres in the anaerobic environment (Lawson et al., 2022). Indeed, if a muscle group is exercised to fatigue, the former mechanism (i.e., metabolic stress) eventually makes the latter mechanism possible (i.e., mechanical tension) (Lawson et al., 2022; Wernbom and Aagaard, 2020), providing a tandem for maximising endocrine responses (Lawson et al., 2022; Cook et al., 2014; Madarame et al., 2008; Jakobsson et al., 2021; Bartolomei et al., 2018; Rahimi et al., 2010). This is supported by findings showing that fatigue-provoking low-load exercise effectively replicates the endogenous production of key anabolic and stress-related factors seen with heavier loads, including the vascular endothelial growth factor and nitric oxide (Hjortshoej et al., 2023). On the other hand, high resistance exercise with longer breaks is limited to the mechanical tension and accompanying molecules (Linnamo et al., 2005), while it fails to perturb the intracellular environment (Hultman and Greenhaff, 1991). Thus, muscle satellite cells, pro-inflammatory cytokines, and immune cells are stimulated to a greater extent by light-to-moderate resistance training in close proximity to failure compared to traditional strength training with heavy loads (Weakley et al., 2023; Rossi et al., 2018). As a result, multiple remodelling factors are released into the bloodstream including ROS, inflammatory markers, microRNAs, and other myokines (Zunner et al., 2022; Wang et al., 2018; Ferraro et al., 2014; Patterson et al., 2013) (Table 1).

**TABLE 1:** Comparison of two different resistance exercise modalities taken to (near) the failure points.

Characteristic	Heavy resistance (1–5 repetitions^a^)	Ligh-to-moderate resistance (>8 repetitions^a^)
Metabolic stress	Low	High
Mechanical tension	High	High
Motor unit recruitment process	Immediate recruitment of high-threshold type 2 fibres	Gradual comprehensive recruitment of type 2 fibres
Fatigue cause	High force demand on type 2 fibres	Accumulated metabolic byproducts leading to high force demand on type 2 fibres
Primary energy system	Phosphagen	Glycolytic/Oxidative
Time to failure	Short	Long
Anabolic pathway signalling (IGF-1/PI3K/Akt/mTOR)	High	High
Satellite cell activity	High	High
Hypertrophy and strength improvements	High	High
Endurance improvements	Low	High
Endocrine factors release	Low	High

^a^ Resistance assessed based on Schoenfeld et al. (2021), although it should be noted that augmented local metabolic stress likely requires the combination of high repetition number (i.e., time under tension) and incomplete recovery breaks.

IGF-1, insulin-like growth factor 1; PI3K, Phosphoinositide 3-kinases; Akt, Protein kinase B; mTOR, mechanistic target of rapamycin.

One important molecule associated with metabolic demands but not with mechanical tension *per se* is lactate, a myokine with autocrine, paracrine and endocrine functions (Lawson et al., 2022; Brooks et al., 2023; Brooks et al., 2022a). Once the lactate production surpasses cellular capacity, it is shuttled into the bloodstream from where it affects multiple tissues and hormones (Brooks et al., 2023; Huang et al., 2021; Nalbandian and Takeda, 2016). Systemic blood lactate levels consistently rise following BFR resistance training with low loads (Fujita et al., 2007; Li et al., 2023), and the associated drop in pH stimulates sympathetic nerve activity, enabling the secretion of growth hormone (Godfrey et al., 2009). Although the LMS triggers a significant release of other anabolic hormones as well (Kraemer and Ratamess, 2005; Fujita et al., 2007; Seo et al., 2016; Manini et al., 2012), the association between testosterone and lactate accumulation is unclear (Walker et al., 2011; Weakley et al., 2017). This could be due to the higher intensity thresholds needed for testosterone [(≥75% of one repetition maximum (1-RM)] (Rahimi et al., 2010; Linnamo et al., 2005) compared to lower resistances (∼60% of 1-RM) (Weakley et al., 2017). In the absence of BFR, regional metabolic perturbations could be provoked by the activation of large muscle groups in close proximity to each other, utilising light-to-moderate loads (Kelleher et al., 2010; Realzola et al., 2022) and short breaks (Kraemer and Ratamess, 2005; Senna et al., 2022; Takarada and Ishii, 2002; Schoenfeld, 2011; Krzysztofik et al., 2019). For example, reciprocal supersets (i.e., paired exercises for agonist and antagonist muscles performed without rest in between) have demonstrated greater effects on energy expenditure, blood lactate levels, and post-exercise oxygen consumption compared to traditional sets with longer rest periods (Zhang et al., 2025; Kelleher et al., 2010; Realzola et al., 2022). In these studies, exercises were paired for the muscles from the opposite side (e.g., leg extension and leg curl) with the equated volume between the training groups (Kelleher et al., 2010; Realzola et al., 2022). Notably, a study that compared supersets involving three consecutive exercises, two consecutive exercises, and a traditional exercise protocol resulted in significantly higher lactate concentrations for the superset groups, particularly for those with more exercises in a row (Weakley et al., 2017). Moreover, supersets involving exercises for the same muscle group (i.e., flat bench press immediately followed by the incline bench press), have been shown to exhibit similar lactate concentration as observed in “pre-exhaustion” and “forced repetitions” groups despite lower total training volume (Wallace et al., 2019). In contrast, a study that used elastic bands to train elbow flexors and extensors found no significant differences in metabolic and mechanical stress markers between reciprocal supersets and traditional 1-min rest intervals (Fink et al., 2021). This discrepancy was likely due to insufficient exercise intensity and the absence of local fatigue in the trained participants (Kraemer and Ratamess, 2005). Therefore, high volume of fatiguing exercises seems necessary to elicit substantial metabolic and hormonal systemic responses (Curovic et al., 2024).

In summary, it appears that LMS can be induced in two ways – either via high volume of fatiguing exercises using low-to-moderate loads and short breaks (Schoenfeld et al., 2017; Fink et al., 2021; Lasevicius et al., 2022; Lacerda et al., 2016), or by BFR applied to the limbs allowing for lighter loads to efficiently facilitate the hypoxic environment (Lixandrão et al., 2018; Hwang and Willoughby, 2019; Krzysztofik et al., 2019). These anaerobic conditions are crucial for the production of metabolic byproducts that drive the adaptations leading to improved strength and hypertrophy (Sødal et al., 2023; Keskin et al., 2025; Kurobe et al., 2015), muscular endurance (Goto et al., 2005; Keskin et al., 2025; Aguiar et al., 2015), cardiometabolic health (Feng et al., 2024; Martin et al., 2022; Saatmann et al., 2021; Hjortshoej et al., 2023; Acosta-Manzano et al., 2020; Zhou et al., 2024; Edwards et al., 2022) and systemic fat tissue reduction (Ramírez-Campillo et al., 2013).

## 3 Local metabolic stress and functional adaptations

### 3.1 Local metabolic stress and endurance

Endurance can be defined as an ability to sustain physical exertion over time (Hughes et al., 2018; Schoenfeld et al., 2021). Aerobic endurance exercise relies on oxygen utilisation for energy production, typically involving whole-body movements like running (Patel et al., 2017). Anaerobic (lactic) endurance, on the other hand, occurs when exercise is performed in the absence of sufficient oxygen supply (Patel et al., 2017; Poole et al., 2021), training the body to withstand the metabolic stress (Fiorenza et al., 2018; Patel et al., 2017; Poole et al., 2021; Gastin, 2001; Haugen et al., 2021). This type of endurance can be trained via high-intensity whole-body movements (Poole et al., 2021; Haugen et al., 2021), or via fatiguing muscle contractions imposed on a targeted muscle group (i.e., muscular endurance) (Fisher J et al., 2020; Hackett et al., 2022). Crucially, from the perspective of systemic *versus* local adjustments, both aerobic and anaerobic whole-body trainings predominantly drive cardiorespiratory adaptations (Farrell et al., 2018; Patel et al., 2017; Poole et al., 2021; Haugen et al., 2021), whereas local anaerobic (i.e., muscular) endurance training primarily imposes muscular adaptations (Farrell et al., 2018; Acosta-Manzano et al., 2020; Fisher J et al., 2020; Hackett et al., 2022). Hence, to differentiate between these categories, they can be referred to as either “systemic aerobic endurance,” “systemic anaerobic endurance” or “local anaerobic (i.e., muscular) endurance.” A challenge emerges, however, if an individual trains multiple upper and lower body muscle groups within the same session following the principles of muscular endurance training guidelines (Triplett and Haff, 2015). In this case, the session could become reliant on the cardiorespiratory system to deal with the energy needs and limit the capacity of separate muscles to perform due to the development of supraspinal fatigue (Taylor et al., 2016; Tanaka and Watanabe, 2012). In fact, it can be proposed that LMS could be strategically manipulated to either optimise local anabolic pathways, enhancing the local muscle growth, strength and muscular endurance (Schoenfeld, 2013), or to facilitate systemic aerobic adaptations by being provoked in separate muscle regions interchangeably, thereby requiring notable post-exercise oxygen consumption (Greer et al., 2021). A meta-analysis comparing HIIT protocols with and without BFR showed that BFR augmented both peripheral and central physiological adaptations, thereby enhancing not only muscular endurance but also the aerobic capacity (Yin et al., 2025). However, while these improvements were likely driven by the elevation of LMS facilitating increases in capillarization, mitochondrial density, cardiac output, and acid-buffering capacity (Yin et al., 2025), the included studies primarily employed intensive running or cycling configurations rather than resistance-based protocols that are less likely to elicit substantial heart rate alterations.

Although vague in its definition, muscular endurance is inherently anaerobic and focuses on repetitive contractions against resistance in hypoxic environment associated with the LMS (de Freitas et al., 2017; Kahraman and Varol, 2024; Schoenfeld et al., 2021; Hackett et al., 2022; Deschenes and Kraemer, 2002). In this context, it must be acknowledged that this type of local endurance encompasses not only the capacity to sustain prolonged efforts against light loads (Schoenfeld et al., 2021) (i.e., “muscular endurance”), but also against moderate loads (i.e., “strength-endurance”) (Fisher J et al., 2020), including the ability to perform with incomplete recovery between sets as well as to complete multiple high-volume sets (Zhang et al., 2025; Fink et al., 2021; Triplett and Haff, 2015). It is, therefore, nearly impossible to establish universal guidelines for training this ability based solely on the number of repetitions or rest durations. That said, standard recommendations differentiate between protocols for muscular endurance and hypertrophy, with the former involving shorter rest intervals (30–60 s), and higher repetition ranges (10–25), and the latter involving longer rest intervals (1–3 min) and fewer repetitions (8–12) (American College of Sports Medicine, 2021). However, hypertrophy can occur equally well with “muscular endurance training” (Schoenfeld et al., 2017; Lasevicius et al., 2022; Fink et al., 2018; Fink et al., 2017), suggesting that muscular endurance could also benefit from “hypertrophy training” (Aguiar et al., 2015). For instance, performing a single exhaustive set at 20% of 1-RM prior to traditional hypertrophy sessions has been shown to significantly enhance both muscular endurance and muscle growth (Aguiar et al., 2015). Furthermore, a systematic review on advanced resistance training techniques indicated that establishing specific guidelines for volume, intensity of effort, and frequency is challenging, as each of these methods appears to maximise muscle responses through the combined effects of mechanical and metabolic stress (Krzysztofik et al., 2019).

It would be unrealistic to expect significant aerobic benefits from local anaerobic conditions due to the cardiorespiratory system not being a primary limiting factor following this type of training (Acosta-Manzano et al., 2020). Notably, however, there are some findings suggesting that an adequate local anaerobic stimulus could simulate exercise modalities that lead to aerobic improvements by imposing significant demands on the cardiorespiratory system (Zhou et al., 2024; Hong et al., 2024). This was illustrated in a study by Hong et al. (2024) which showed that resistance exercise involving large muscle groups such as squats could promote aerobic gains through increased oxygen demands when multiple sets of 10 repetitions were performed (Hong et al., 2024). Similarly, greater metabolic demands in superset configurations have been linked to the elevated post-exercise oxygen consumption (Kelleher et al., 2010; Realzola et al., 2022), potentially triggering aerobic advancements. In agreement, a systematic review with meta-analysis revealed that both muscular endurance and hypertrophy-focused training led to improved cardiorespiratory fitness (Acosta-Manzano et al., 2020). These findings collectively show that when large muscle groups are exercised with a LMS approach, they can contribute to systemic aerobic adaptations, although the extent and consistency of those effects may be limited (Farrell et al., 2018). For example, complementing an aerobic endurance programme with local muscular endurance exercise did not enhance VO2 max in active individuals, though it did increase VO2 max at the onset of blood lactate accumulation (Farrell et al., 2018).

In summary, repeatedly imposed LMS can lead to improved muscular endurance, a term that should be used to describe resistance to fatigue not only with light loads, but also with moderate loads (i.e., strength-endurance) concerning both the contractions to failure and the ability to perform with incomplete breaks. Its role in promoting systemic (aerobic) endurance may be less impactful, but not without positive effects for recreational populations, particularly if different body regions are simultaneously trained.

### 3.2 Local metabolic stress indirectly leads to hypertrophy and strength with decreasing loads

Complex mechanisms are being increasingly acknowledged as important mediators of hypertrophy, including mTORC1 signaling, myonuclear accretion (i.e., fusion of satellite cells with the existing muscle fibre), microRNA stimulation, enhanced angiogenesis (i.e., formation of new blood vessels), gene expression, mitochondrial biogenesis and similar (Roberts et al., 2023). Unfortunately, the extent to which these mechanisms can be triggered directly by the LMS is unclear. As previously noted, when resistance exercise with light and moderate loads is performed with the close proximity to failure, it can lead to equal hypertrophy gains as exercise with heavier loads (Lixandrão et al., 2018; Keskin et al., 2025; Schoenfeld et al., 2017; Zhang et al., 2025; Farup et al., 2015; Lim et al., 2019) and comparable strength gains as seen with traditional strength training (Weakley et al., 2023; Lim et al., 2019; Grønfeldt et al., 2020). The consistent finding that BFR training with very light loads (∼30% of 1-RM) achieves similar results (Lixandrão et al., 2018; Farup et al., 2015), jointly underlines the contribution of metabolite accumulation to those outcomes (Pearson and Hussain, 2015). The role of the LMS for hypertrophy and strength adaptations may be quite simply explained via increased neural drive to the exercising muscle’s type 2 fibres (Conwit et al., 1999; Morton et al., 2016). As the fatigue develops in oxidative fibres, maintaining the same effort requires recruiting additional motor units thereby activating high-threshold fibres (Sundstrup et al., 2012). This expanded recruitment allows more fibres to experience mechanical tension necessary for hypertrophy (Lawson et al., 2022; Wackerhage et al., 2019) under the condition that repetitions are performed until near the failure points (Schoenfeld et al., 2017; Robinson et al., 2024; Lasevicius et al., 2022). Hence, significant hypertrophy gains with light loads would be impossible to occur if there was no metabolic stress to facilitate this process (Hwang and Willoughby, 2019; Wernbom and Aagaard, 2020; Krzysztofik et al., 2019). On the other hand, strength, being primarily a neuromuscular quality (Triplett and Haff, 2015), may depend more on the intensified voluntary activation with high-force, low-fatigue contractions compared to the high-volume of fatiguing sets (Robinson et al., 2024), as the latter may reduce the efficiency of rapid motor unit recruitment in the subsequent sets (Suchomel et al., 2018).

It should be noted that the occurrence of fatigue aligned with an emphasised LMS may diminish the additional hypertrophic stimuli in the later sets (Nordlund et al., 2004). The main critics of this training modality point out that shorter rest periods and exercise to failure might reduce the total training volume, which is a valid concern. However, since volume is defined as the number of sets multiplied by the number of repetitions, fatigue in the exercising muscles can be manipulated by using lighter loads later in the session (Iversen et al., 2021). In fact, repetitions with submaximal loads performed under the hypoxic local environment yield greater hypertrophic response compared to those performed short of inducing fatigue despite the same total volume (Karsten et al., 2021). Thus, shortening rest periods can be an effective method for creating and maintaining local anaerobic conditions, allowing for more stimulating repetitions. In agreement, time-efficient training strategies with lighter resistance and shorter breaks lead to gains equal to traditional strength training (Zhang et al., 2025; Krzysztofik et al., 2019; Iversen et al., 2021). For example, Farup et al. (2015) examined muscular adaptations following resistance programme with loads around 40% of 1-RM involving 4 sets of unilateral dumbbell curls to failure with the only difference between arms being BFR applied to one limb. Despite the BFR arm performing less repetitions per set (resulting in lower total volume and training time), muscle size increased similarly in both limbs after the programme (Farup et al., 2015). That said, it should be noted that resistance below 20% of 1-RM may be suboptimal (Ozaki et al., 2016; Lasevicius et al., 2018) due to the inefficiency of the muscle to trap metabolites inside the cell (de Freitas et al., 2017; Lasevicius et al., 2018). In this scenario, employing a superset configuration may be helpful to limit the recovery of oxygen availability in the exercising muscles (Weakley et al., 2017; Kelleher et al., 2010; Realzola et al., 2022; Fink et al., 2018). It is also important to highlight that exercising to failure does not appear superior to stopping just short of failure (Grgic et al., 2022), though close proximity looks essential with low loads (Lasevicius et al., 2022). It would be interesting to examine whether the combination of the reciprocal superset and drop set concept (e.g., fatiguing repetitions including a moderate load for agonist followed by the moderate load for antagonist + light load for the same agonist + light load for the same antagonist), followed by longer inter-superset periods, could augment the hypertrophic stimulus in the selected body region and result in the completed session within a shorter timeframe. Notably, tolerance to this method could vary based on the training experience, making it more suitable for highly trained individuals.

#### 3.2.1 Local metabolic stress facilitates vertical strength transfer

Although endogenous hormonal elevations may play a less significant role for the facilitation of strength and hypertrophy than previously believed (Loenneke et al., 2011; West et al., 2010), the role of metabolites should not be dismissed (Lawson et al., 2022; Curovic et al., 2024). Indeed, studies indicate that intramuscular hypoxia manifested by elevated lactate concentrations, oxygenated hemoglobin (Goto et al., 2005) and growth hormone (Kurobe et al., 2015) directly stimulates muscular hypertrophy (Goto et al., 2005; Kurobe et al., 2015; Aguiar et al., 2015), and that external infusion of lactate in mice results in molecular signaling linked to hypertrophy independent of an exercise stimulus (Cerda‐Kohler et al., 2018). Interestingly, locally produced endocrine factors associated with metabolic stress may enter the circulation and contribute to the whole-body anabolic adaptations via neurophysiological mechanisms (Brooks et al., 2023; Curovic et al., 2024; Nalbandian and Takeda, 2016; Quistorff et al., 2008). These circulating exerkines and exosomes include lactate (Brooks et al., 2023; Nalbandian and Takeda, 2016; Quistorff et al., 2008), which can promote neuroplasticity and reduce intracortical inhibition, thereby increasing the firing rate of neurons (Huang et al., 2021; Xue et al., 2022; Andrews et al., 2020), followed by ROS (Centner et al., 2018), microRNA (Domańska-Senderowska et al., 2019), and follistatin (Bagheri et al., 2019; Tortoriello et al., 2001), a glycoprotein that suppresses muscle-wasting myostatin (Laurentino et al., 2012). For example, when legs are exercised with the goal of provoking high metabolic response sufficient to perturb systemic circulation, upper body muscles trained separately in the session experience amplified strength and hypertrophy adaptations (May et al., 2018; Cook et al., 2014; Rønnestad et al., 2011; Madarame et al., 2008; Bartolomei et al., 2018; Hansen et al., 2001), though not in all instances (Jakobsson et al., 2021; West et al., 2010). While findings on strength augmentation in the muscle trained before resistance exercise-induced LMS from another body region are conflicting (May et al., 2018; West et al., 2010; Hansen et al., 2001), other studies consistently suggest a strength-enhancing effect when a targeted muscle is trained subsequently to the elevated LMS (Spiering et al., 2009; Rønnestad et al., 2011; Spillane et al., 2015). For example, it has been demonstrated that upper body resistance training session with high metabolic and hormonal perturbations effectively potentiated androgen receptors in the subsequently exercised lower body (Spiering et al., 2009; Spillane et al., 2015), implying a strength-augmenting effect for the leg muscle (Kraemer and Ratamess, 2005). Remarkably, this type of metabolically challenging upper body resistance training could offer protection for the leg muscle morphology and cycling power when positioned after exhaustive endurance running sessions on the same day (Kraemer et al., 1995). This phenomenon, recently named as “vertical strength transfer” (Curovic et al., 2024), explains that strength-trained muscles (i.e., high resistance, low volume) from one body region (e.g., upper body) may be positively influenced by high-volume metabolic exercise from remote body regions (e.g., lower body) through the triggered differential intramuscular signaling, leading to greater hypertrophy and muscle remodeling (Kraemer et al., 1995; Moberg et al., 2021; Verney et al., 2008; Verney et al., 2006; Abaïdia et al., 2017). These neurophysiological effects are proposed to be enabled by the endocrine factors associated with the LMS (Spiering et al., 2009; Cook et al., 2014; Spillane et al., 2015; Madarame et al., 2008; Hansen et al., 2001; Kraemer et al., 1995) (Table 2). One possible explanation for the centrally mediated benefits of increased metabolic stress on strength and hypertrophy adaptations may involve a shift in neuronal firing towards preferential activation of type 2 muscle fibres even in remote muscle groups. This is supported by the study showing enhanced strength and muscular endurance in the leg muscles trained under systemically induced anaerobic conditions by normobaric hypoxic exposure (Manimmanakorn et al., 2013). This study revealed that resistance training with light loads under BFR was not superior compared to the group with systemically deprived oxygen availability because both led to substantial improvements compared to control (Manimmanakorn et al., 2013).

**TABLE 2:** Local metabolic stress and circulating endocrine factors influencing remote muscles via “vertical strength transfer” [table adapted by Curovic et al. (2024)]

Study	Training duration	Study design/groups	Outcome measures of interest	Key findings	Systemic influence by local metabolic stress
Kraemer et al. (1995)	12 weeks, 4x per week	*Between subject design* EG: 40 min running sessions 2x/week; 200–800 m interval sessions 2x/week USEG: same running sessions followed by resistance trainings for all UB muscle groups with the combination of high volume and moderate/high resistance (5–10 RM) using supersets and short rests (1–3 min)	- 1-RM bench press and 1-RM military press (kg) - Wingate anaerobic test (W) - Vastus lateralis muscle fiber morphology (μm^2^) and leg muscle fiber area -Testosterone (nM) and cortisol (nM)	UB resistance training prevented leg power loss evident in EG UB resistance training prevented significant decrease in type I and type IIc fibre area of vastus lateralis muscle evident in EG UB resistance training prevented cortisol increase seen in EG	Yes, from UB to LB
Hansen et al. (2001)	9 weeks, 2x/week	*Between subject design* Unilateral arm training standardised for both groups Leg resistance trainings organised with 4 sets of leg press, 8–12 repetitions and 1 min rest periods for the intervention group	- Elbow flexors MVIC (Nm) - Testosterone (nM), cortisol (nM), and growth hormone (mg/L)	Significant increases in isometric strength of both the trained arm (37%) and control arm (10%) for the unilateral arm followed by leg training group Cortisol, testosterone, and growth hormone higher in leg training group	Yes, from LB to UB
Madarame et al. (2008)	10 weeks, 2x/week	*Between subject design* Unilateral arm training standardised for both groups Leg resistance training standardised for both groups with 3 sets of knee extension and knee flexion exercises at 30% of 1-RM; repetitions: 30 + 20 + 20 with 30 s rests BFR applied to legs in the intervention group	- Maximal isometric elbow flexors torque (Nm) and elbow flexors CSA (cm^2^) - Noradrenaline (nM), testosterone (nM), and growth hormone (ng/mL)	Significant hypertrophy and isometric strength improvement in the trained arm (*p* < 0.05) only for the BFR-leg training group No changes in the untrained arm Noradrenaline concentration-time curve significantly higher in the BFR-leg training group (182.2 ± 79.0 nM for 30 min) compared to the normal exercise group (79.4 ± 16.0 nM for 30 min) (*p* < 0.05)	Yes, from LB to UB
May et al. (2018)	7 weeks, 3x/week	*Between subject design* Unilateral arm training standardised for both groups Leg resistance training standardised for both groups with 4 sets of knee extension and knee flexion exercises at 30% of 1-RM; repetitions: 30 + 15 + 15 + 15 with 30 s rests BFR applied to legs in the intervention group	- 1-RM elbow flexion (kg) and elbow flexors CSA (cm^2^)	Higher increases in trained arm strength for leg-BFR group (2.5 ± 0.4 kg vs. 0.8 ± 0.4 kg) No effects on muscle CSA	Yes, from LB to UB
Cook et al. (2014)	3 weeks, 3x/week	*Between subject design* Whole-body training standardised for both groups including leg squat, bench press, and weighted pull-up at 70% of 1-RM. Five sets of 5 repetitions were performed with 90 s rest between sets and 3 min between exercises BFR applied to legs in the intervention group	- 1-RM bench press (kg) -Testosterone (ng/mL) and cortisol (ng/mL)	Significantly greater improvements in bench press for leg-BFR group (5.4 ± 2.6 vs. 3.3 ± 1.4 kg; *p* = 0.0044, 1.4% ± 0.8%) Significant associations between mean acute salivary testosterone response and bench press strength (*r* = 0,45, *p* = 0,0233)	Yes, from LB to UB
Ampomah et al. (2019)	10 weeks, 2x/week	*Between subject design* Whole-body training standardised for both groups including leg extension, plantar flexion and elbow flexion with 3 sets at 25% of the MVIC to failure and 30–60 s rests between sets. Subsequently, 3 sets of trunk extension at 25% of the MVIC were performed with 15 repetitions and 30–60 s rests BFR applied to all limbs in the intervention group	- Trunk extensor MVIC (Nm), trunk endurance (s), and erector spinae CSA (cm^2^)	No significant changes in improvements between the groups	Equal improvements for trunk when all limbs were trained to failure regardless of the BFR
West et al. (2010)	15 weeks, 2–3x/week each arm	*Within subject design* Unilateral arm training standardised for both arms One arm followed by leg training including 5 sets of 10 repetitions using leg press and 3 sets of 12 repetitions using leg extension/leg curl “supersets” at ∼90% of 10-RM	- 1-RM and 10-RM (kg), and MVIC (Nm) of elbow flexors - Testosterone (nM), growth hormone (μM), and IGF-1 (nM)	Significantly elevated endogenous hormones after leg trainings (*p* < 0.001) did not result in an increased arm hypertrophy response or strength after the programme	No influence from LB to UB
Rønnestad et al. (2011)	11 weeks, 2x/week each arm	*Within subject design* Unilateral arm training standardised for both arms One arm preceded by leg training including leg press, knee extension and knee flexion with 3 sets at 10-RM using 60–90 s rest between sets	- 1-RM elbow flexion (kg) and elbow flexors CSA (cm^2^) -Testosterone (nM), growth hormone (mlE/L), and cortisol (mlE/L)	Strength improvements higher after leg training (21%) than without (14%) Significant increases in the trained arm’s largest area after leg training (*p* < 0.001) Significantly increased serum testosterone and growth hormone levels after leg training (*p* < 0.05)	Yes, from LB to UB
Jakobsson et al. (2021)	10 weeks, 3x/week	*Between subject design* UB strength training standardised for both groups (3 sets, 4–6 repetitions at 80%–90% of 1-RM, 3 min rest) Leg resistance training organised either with identical approach as UB (control group) or with 10–15 reps at 60%–70% of 1-RM with 60 s rests (intervention group); both groups used leg press, leg extension and leg curl exercises	- 1-RM bench press and 1-RM lat pull-down (kg) - Testosterone (nmol/L) and growth hormone (ug/L)	No significant differences in UB strength improvements Greater increase in growth hormone for high-volume group	No influence from LB to UB
Bartolomei et al. (2018)	6 weeks, 4x/week	*Between subject design* UB strength training standardised for both groups (5 sets, 4-5 repetitions at 88%–90% of 1-RM, 2 min rests) Leg resistance training organised either with identical approach as UB (control group) or with 10–12 reps at 65%–70% of 1-RM with 60 s rests (intervention group); both groups used squats, leg press, leg extension, leg curl and calf raise machine exercises	- 1-RM bench press (kg), 50% of 1-RM ballistic bench press (W), and arm muscle size (cm)	More significant hypertrophy for arm muscle area (*p* = 0.046), more significant UB strength increase (*p* = 0.007), and more significant UB power expression (*p* = 0.011) with high volume leg training approach	Yes, from LB to UB
Spiering et al. (2009)	Acute study protocol	*Within subject design* Leg strength training standardised in both protocols with 5 sets of 5-RM knee extensions and 3 min rests In one protocol, leg training was preceded by UB resistance session that included 4 sets of 10-RM bench press, bench row, and seated overhead press to failure with 2 min rests	-Testosterone (nM/L) and muscle androgen receptors (au)	Significantly increased serum testosterone after UB training vs. leg training only (*p* = 0.01–0.04; effect size = 0.63–1.03). Testosterone area under the curve 14% greater (*p* < 0.01; effect size = 0.60)	Yes, from UB to LB
Spillane et al. (2015)	Acute study protocol	*Within subject design* Leg strength training standardised in both protocols with 5 sets of 5-RM knee extensions and 3 min rests In one protocol, leg training was preceded by UB resistance session that included 4 sets of 10-RM bench press, bench row, and seated overhead press to failure with 2 min rests	-Testosterone (nM/L) and β-Catenin (Abs/ug)	No significant difference in testosterone levels between the groups. β-Catenin significantly greater after UB training vs. leg training only at 3 h (*p* = 0.001) and 24 h (*p* = 0.001) post-exercise	Yes, from UB to LB

UB, upper body; LB, lower body; CSA, cross-sectional area; BFR, blood-flow restriction; IGF-1, insulin-like growth factor-1; RM, repetition maximum; MVIC, maximal voluntary isometric contraction; EG, endurance training group; USEG, upper body strength and endurance training group; CG, control group.

In summary, training volumes concerning stimulating repetitions with the close proximity to failure may be the most important variable for hypertrophy and strength improvements with decreasing loads (Schoenfeld et al., 2017; Lasevicius et al., 2022; Lim et al., 2019; Morton et al., 2016; Karsten et al., 2021; Dankel et al., 2017; Ampomah et al., 2019), although reaching an absolute failure does not seem to be necessary (Grgic et al., 2022). Short rest periods, especially when using a very light resistance, or the strategic use of supersets or BFR, may be utilised to magnify the hypoxic local conditions that lead to greater levels of mechanical tension and local muscle growth (Weakley et al., 2017; Realzola et al., 2022; Fink et al., 2018; Fink et al., 2017). Strikingly, these conditions also contribute to anabolic and anti-catabolic adaptations in distant muscle cells, suggesting beneficial systemic influence on a whole-body level (Curovic et al., 2024). LMS-mediated strength and hypertrophy adaptations are likely explained via well-established physiological and biomechanical models such as the size principle and force-velocity relationship (i.e., slowing down of contractions near the failure point with high forces generated by high-threshold fibres) (Lawson et al., 2022; Wernbom and Aagaard, 2020; Schoenfeld, 2013; Myrholt et al., 2023). Nevertheless, maximal strength improvements still need to primarily rely on heavier loads (Schoenfeld et al., 2017), although untrained and recreationally active individuals could experience similar strength gains with lighter resistance under the aforementioned conditions (Grgic et al., 2018; Grønfeldt et al., 2020).

## 4 Local metabolic stress, immune system and health

**FIGURE 1:**
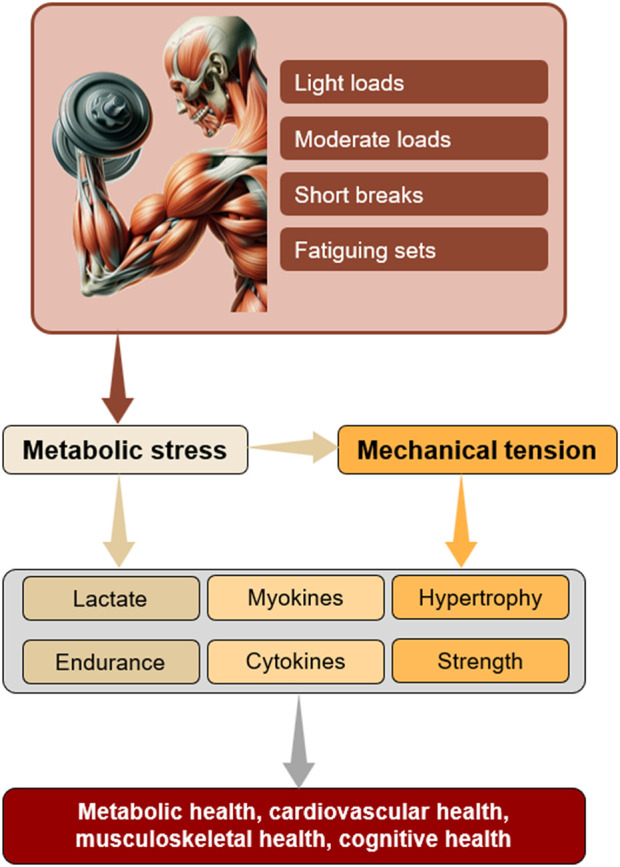
Proposed mechanisms through which locally induced metabolic stress promotes a broad spectrum of beneficial adaptations. Note that blood flow restriction enhances metabolic conditions, enabling the use of lighter loads.

Life-extending practices often involve controlled stressors that enhance resilience and adaptability through a process known as allostasis (Calabrese et al., 2024; Zhu et al., 2022). For instance, when the body is exposed to extreme cold or heat, it triggers the production of shock proteins and other molecules that protect cells and enhance their survival mechanisms (Patrick and Johnson, 2021; Kunutsor et al., 2024). Exercise-induced allostasis highlights that beneficial adaptations occur within a threshold of stress tolerance (Pearson and Hussain, 2015), explaining why moderate endurance activities tend to have linear associations with lifespan, while excessive number of long and intense whole-body endurance sessions does not always lead to better outcomes (O’Keefe et al., 2023; Franklin et al., 2020). Local anaerobic endurance training, however, may serve as a potent, yet manageable stimulus for several benefits associated with exercise stress. As suggested in previous sections, it could trigger both the mechanical stress pathways (associated with traditional strength training) and metabolic stress pathways (associated with endurance training) (Martin et al., 2022) that jointly lead to local and systemic endurance (Fink et al., 2021; Acosta-Manzano et al., 2020), strength and muscle growth (Lixandrão et al., 2018; Schoenfeld et al., 2017; Martin et al., 2022; Grønfeldt et al., 2020), and preserved health (Feng et al., 2024; Feng et al., 2024; Bemben, 1998; Chow et al., 2022; Saatmann et al., 2021; Zhu et al., 2022) (Figure 1). Indeed, this type of exercise may potently stimulate a comprehensive release of various myokines and exosomes (Goto et al., 2005; Coffey and Hawley, 2007; Hjortshoej et al., 2023; Huang et al., 2024; Guo et al., 2023) that have wide-ranging effects on immune system regulation (Afzali et al., 2018). Hence, it is somewhat surprising that, while strength (García-Hermoso et al., 2018; Li et al., 2018) and aerobic capacity (Strasser and Burtscher, 2018; Leslie et al., 2023) are well researched contributors to longevity, the associations concerning the local muscular (anaerobic) endurance capacity remain largely underexplored (Bemben, 1998; Acosta-Manzano et al., 2020) despite the noted potential (Goto et al., 2005; Kahraman and Varol, 2024; Farrell et al., 2018; Acosta-Manzano et al., 2020; Ramírez-Campillo et al., 2013; Wang et al., 2023).

### 4.1 Reactive oxygen species, cytokines and myokines

LMS-inducing exercise such as seen with BFR training, triggers adaptive mechanisms linked to various physiological effects (Hjortshoej et al., 2023). These involve enhanced muscle fibre recruitment, proliferation of myogenic satellite cells and angiogenesis, as well as acute hormonal, immune, and oxidative stress responses (Goto et al., 2005; Hjortshoej et al., 2023). All those effects synergistically contribute to exercise’s various benefits, including the mitigation of post-traumatic muscle atrophy (van Melick et al., 2016; Lepley et al., 2020), enhancement of neuromuscular activity (Curovic et al., 2024; Bartolomei et al., 2018; Cook et al., 2013), pain reduction (Liu et al., 2024; Hughes and Patterson, 2020; Korakakis et al., 2018), and increased bone mineral density (Jack et al., 2023). Several therapeutic myokines and exerkines are identified as a response to resistance training stimuli (Coffey and Hawley, 2007; Hjortshoej et al., 2023). Amongst them, ROS and cytokines stand out for their immune-regulating characteristics (Ferraro et al., 2014; Nelke et al., 2019). Indeed, findings suggest that exercise-provoked ROS and proinflammatory cytokines, otherwise damaging molecules, can actually promote positive tissue remodeling, supporting the overall immunity (Ferraro et al., 2014; Nelke et al., 2019). For example, taking antioxidant supplements post-training diminishes the beneficial effects of ROS-mediated cell signaling (Merry and Ristow, 2016), highlighting their contribution to improved endogenous antioxidant defenses. The importance of LMS for ROS production has been proposed by findings that highlight low-load BFR training superiority in elevating systemic ROS levels compared to traditional low-load or high-load exercise routines (Martin et al., 2022; Hjortshoej et al., 2023; Centner et al., 2018). Nonetheless, it appears that moderate resistance exercise, which generates greater mechanical stress, stimulates ROS production more effectively than light resistance without BFR (Goldfarb et al., 2008), likely via inflicted muscle damage (Owens et al., 2019; Powers et al., 2011). Although muscle damage and metabolic stress differ in their mechanisms (Martin et al., 2022), training to failure with minimal rest periods could be accompanied with some degree of microtears in the exercising fibres (Martin et al., 2022; Senna et al., 2022). Depending on the severity of this, the muscle’s recovery capacity could be impaired, though some portion of damage can actually benefit the body’s adaptive mechanisms (de Lemos Muller et al., 2019; Owens et al., 2019; Tee et al., 2007; Leal et al., 2018; Armstrong et al., 2012).

Metabolic stress may be one of the key factors behind the production of cytokines, the inflammatory markers associated with exercise’s health effects (Nelke et al., 2019). Interleukin (IL)-6 is released from muscles in response to an energy deficit (Kistner et al., 2022), with its levels rising in proportion to the exercise intensity and duration (Raschke and Eckel, 2013). As an energy sensor (Docherty et al., 2022), IL-6 is upregulated by low glycogen levels (Keller et al., 2001), playing a key role in the body’s metabolic responses (Docherty et al., 2022). Initially thought to be activated only by muscle damage (Patterson et al., 2013), recent studies show inconsistent findings regarding its stimulation with LMS being a potential candidate (Docherty et al., 2022; Pedersen and Febbraio, 2008). While IL-6 is generally a pro-inflammatory cytokine, it exhibits anti-inflammatory effects when stimulated by physical training (Huh, 2018; Muñoz-Cánoves et al., 2013). In addition to its immune-regulating role, IL-6 can improve systemic glucose and fat metabolism (Steinbacher and Eckl, 2015), restore muscle function, and mobilise satellite cells for reparation (Serrano et al., 2008). In contrast, when the whole body is exposed to a high-intensity training session with prolonged duration, this cytokine can jeopardise the immunity and promote muscle wasting (Muñoz-Cánoves et al., 2013). Thus, a possible alternative to frequent HIIT regimes may be found in exercises that induce stress only locally, helping to preserve IL-6’s immune-protective function. Still, this remains speculative and requires further investigation. Despite other interleukins being less researched (Docherty et al., 2022), a notable potential has also been identified with exercise-provoked IL-7 and IL-15, due to their outstanding role in enhancing the immune system (Nelke et al., 2019; Haugen et al., 2010; Duggal et al., 2018). These cytokines are associated with the restoration of 5′AMP-activated protein kinase (AMPK), a critical regulator of metabolic health (Coffey and Hawley, 2007) that declines with aging (Duggal et al., 2018), as well as with the development and maintenance of immature lymphocytes (Duggal et al., 2018). Further research is required to unveil the extent to which the LMS could affect and promote cytokines’ beneficial influence (Nelke et al., 2019).

Myokines are signaling proteins released by muscle cells in response to exercise (Zunner et al., 2022), playing a crucial role in regulating inflammation, metabolism (Laurens et al., 2020; Huh, 2018), muscle repair (Brandt and Pedersen, 2010), and overall health (Bucarey et al., 2024; Brandt and Pedersen, 2010; Leal et al., 2018; Gomarasca et al., 2020). As discussed in the previous sections, their release may be closely tied to the LMS, stimulated by “hypertrophy training” and “strength-endurance training” (Zunner et al., 2022). However, due to the lack of research on specific resistance exercise differences in augmenting myokine production (Bettariga et al., 2024), it is difficult to assess these associations. One interesting group of myokines modified by resistance exercise involves myostatin, follistatin and decorin (Zunner et al., 2022). Myostatin negatively affects muscle mass (Lee and June 2019), and it seems to be suppressed by resistance training-induced decorin and follistatin (Zunner et al., 2022; Bagheri et al., 2019; Khalafi et al., 2023; Willoughby et al., 2022). A single study that compared low-load BFR exercise to the heavy-load protocol revealed similar downregulation of myostatin in both instances (Laurentino et al., 2012). It may be the case that the suppression of this muscle-wasting signalling factor is aligned with the mechanical tension, and that BFR exercise was simply able to provoke enough (metabolic) stimulus for this to occur. Future research is needed to explore the extent of the regulatory influence exerted by various resistance exercise modes on these myokines, given their significant protective potential. For example, decorin has demonstrated the ability to inhibit angiogenesis and tumorigenesis (Baghy et al., 2020), while follistatin is linked to the healing of muscle injuries (Zhu et al., 2011).

Irisin is a myokine associated with both endurance and resistance exercise (Zunner et al., 2022), strongly corresponding to the AMPK activity and peroxisome proliferator-activated receptor gamma coactivator 1-alpha (PGC-1α) pathways, which are typically activated by endurance training (Zunner et al., 2022; Hjortshoej et al., 2023). Yet, this myokine also correlates with increased muscle mass and strength (Martínez Muñoz et al., 2019; Kim et al., 2016), suggesting a multifaceted beneficial impact. Irisin has demonstrated positive effects on metabolic and musculoskeletal health (Liu et al., 2021), as well as on the enhancement of cognitive function (Islam et al., 2021). To the author’s knowledge, only one study has compared the influence of LMS-inducing exercise (via BFR approach) against traditional strength training, finding greater concentrations in circulating irisin with the former modality (Kraemer et al., 2016). Further research is therefore warranted to explore the mechanisms underlying irisin’s stimulation.

Another myokine associated with both strength and endurance training is a “meteorite-like” molecule that indirectly supports adipocyte browning and mitochondrial health by enhancing immune cell function, while upregulating anti-inflammatory cytokines (Rao et al., 2014). It is closely connected to the PGC1a4, a significant subpart of a large PGC – 1α transcription group that also includes PGC-1α1. Both PGC-1α1 and PGC-1α4 are elevated in the system by the combination of resistance and vigorous aerobic exercise (Moberg et al., 2021; Hansson et al., 2019; Lundberg et al., 2014). While PGC-1α1 responds well to endurance training and improves aerobic adaptations (Agudelo et al., 2019; Mortensen et al., 2006), resistance training yields metabolic benefits via PGC-1α4, a cofactor that enhances glycolysis and ATP production in the muscle (Koh et al., 2022; Neto et al., 2023). PGC-1α group contributes to metabolic health (Neto et al., 2023) and prevention of pathophysiological diseases (Qian et al., 2024), possibly achieved by its overexpression in myotubes promoting fat oxidation and anaerobic glycolysis (Neto et al., 2023). The potential of the LMS to simultaneously target and stimulate the two isoforms of PGC-1α (PGC-1α4 and PGC-1α4) may be related to the dual influence of metabolite accumulation and mechanical tension. This needs to be explored through well-designed training studies targeting the elevation of the LMS and examining its effects on both transcriptional pathways.

Brain-derived neurotrophic factor (BDNF) is produced by various cells in the body (Zunner et al., 2022). When BDNF is stimulated by muscle contractions, it can assist in neuroprotection and prevent neurodegeneration (Palasz et al., 2020). This myokine may be potentiated by the heightened LMS during high-volume resistance exercise protocols. One study has shown that fatigue-provoking training consisting of 3 sets of 10 repetitions with 60-s rest intervals elicited a significantly greater increase (*p* < 0.01) in peripheral serum compared to 5 sets of 5 repetitions with 180-s rests (Marston et al., 2017). Notably, lactate and BDNF changes were positively correlated only after the local strength-endurance protocol (*r* = 0.70; *p* < 0.01), but not following a traditional strength training protocol (*r* = 0.18; *p* = 0.56) (Marston et al., 2017). This suggests that LMS could have played a facilitatory role in both direct and indirect stimulation of BDNF via enhanced conditions for mechanical tension (Lawson et al., 2022). In contrast, a separate study found that neither high-load nor moderate-load resistance training twice a week for 12 weeks affected peripheral growth factors (Marston et al., 2019). The authors proposed that higher frequency of high-volume sessions might be necessary for the enhancement of growth factors (Marston et al., 2019), although achieving this balance may be challenging for recreational individuals. It would be interesting to examine whether BFR could augment the changes seen in BDNF and upregulate its expression in response to resistance training stimulus.

### 4.2 Local metabolic stress and health effects

One of the most significant benefits of LMS may be aligned to improved cardiovascular health (Yang et al., 2019; Devereux, 2010; Zhou et al., 2024; Angelopoulos et al., 2023). Isometric resistance training causes greater blood pressure reductions than any other training modality (Owen et al., 2010; Inder et al., 2016; Edwards et al., 2023), with its intensity and associated local muscle fatigue closely linked to those effects (Devereux, 2010; Owen et al., 2010; Edwards et al., 2022; Inder et al., 2016; Devereux et al., 2012). Ischemic anaerobic conditions during isometric exercises (Robergs et al., 2004) trigger vasodilation and vascular adaptations proposed to enhance blood flow (Edwards et al., 2022). Consequently, the resting systolic blood pressure and mean arterial pressure are significantly reduced over time (Devereux, 2010; Edwards et al., 2022). These reductions are closely connected to the local fatigue measured by peak lactate, highlighting the importance of LMS in promoting hypotensive adaptations (Devereux et al., 2012). Interestingly, a study by Peters et al. (2006) suggested the role of ROS as mediating factors between the therapeutic influence of isometric training intervention for hypertensive adults and the significant blood pressure reductions reported (mean reduction 13 mm Hg, *p* < 0.05) (Peters et al., 2006). The authors attributed this effect to a decrease in oxidative stress (Peters et al., 2006), enabled by the ROS-dependent intracellular signaling that enhances endogenous antioxidant defenses (Sauer et al., 2001). A promising role of LMS to facilitate hypertension improvements was also suggested in a recent systematic review with meta-analysis (Feng et al., 2024) which showed that BFR training was able to significantly reduce blood pressure in middle-aged and elderly women (Feng et al., 2024). Furthermore, potential of the LMS adaptations to preserve cardiovascular health was observed in a longitudinal cohort study of 1,104 adult men, where a significant negative association was reported between the push-up capacity and cardiovascular disease incidence over 10 years (Yang et al., 2019). It is, therefore, reasonable to suggest that fatiguing low-resistance training does not only offer a safe alternative (Miller et al., 2021) to a heavy-resistance training (Martin et al., 2022) and extreme endurance training (O’Keefe et al., 2023; O’Keefe et al., 2015) for individuals with cardiovascular risks, but could also serve as a therapeutical intervention to target hypertensive conditions (Devereux, 2010; Feng et al., 2024).

Lactate is among the main mediators of health-promoting effects by local resistance exercise that triggers its accumulation (Lawson et al., 2022; Fiorenza et al., 2018; Nalbandian and Takeda, 2016; Fujita et al., 2007; Li et al., 2023; Kelleher et al., 2010; Realzola et al., 2022; Devereux et al., 2012). This metabolite acts as a signaling molecule shuttled to distant tissues (Brooks et al., 2023; Brooks et al., 2022a; Nalbandian and Takeda, 2016; Brooks et al., 2022b), where it exerts paracrine effects linked to the endogenous production of growth hormone, testosterone, cortisol, insulin-like growth factor 1, vascular endothelial growth factor, noradrenaline, and nitric oxide (Lawson et al., 2022; Brooks et al., 2023; Farrell et al., 2018; Rønnestad et al., 2011; Godfrey et al., 2009; Hjortshoej et al., 2023; Jakobsson et al., 2021; Cerda‐Kohler et al., 2018). Lactate positively affects glucose metabolism (Saatmann et al., 2021; Acosta-Manzano et al., 2020), bone health (Gomarasca et al., 2020; Liu et al., 2021), and immune cell recruitment (Rossi et al., 2018), potentially even leading to protective effects on cancer occurrence (Zhu et al., 2022). Moreover, lactate is preferentially utilised as an energy resource by brain cells (Quistorff et al., 2008; Xue et al., 2022), where it supports neuroplasticity and cerebrovascular adaptability (Huang et al., 2021; Xue et al., 2022), thereby exerting neuroprotective effects (Bu et al., 2025; Huang et al., 2024; Deng et al., 2024; Vaynman and Gomez-Pinilla, 2006). Emerging evidence suggests that lactate metabolism beneficially links physical activity, gut microbiota and neurodegeneration, highlighting the need for further research to understand those interactions and develop interventions to mitigate the progression of neurological diseases (Rojas-Valverde et al., 2023). An interesting study by Blackmore et al. (2024) found that high-intensity exercise involving anaerobic zones significantly enhanced hippocampal function, preserved cortical volume, and strengthened neural network connectivity in aged individuals (Blackmore et al., 2024). These benefits, which persisted for at least 5 years, were associated with intensity-dependent changes in circulating biochemical markers, highlighting the need to investigate how aerobic-anaerobic transitions contribute to neuroprotection (Blackmore et al., 2024). Furthermore, lactates independently lead to molecular adaptations beneficial for muscle tissues (Cerda‐Kohler et al., 2018), and their rise is associated with the downregulation of ghrelin (McCarthy et al., 2023), a finding that carries important implications for weight loss strategies via appetite suppression. Findings also suggest that LMS could enhance angiogenic gene expression (Larkin et al., 2012) and mitochondrial biogenic mRNA responses (Fiorenza et al., 2018), possibly due to the post-exercise elevations in muscle hemoglobin concentrations (Larkin et al., 2012). Future research is, therefore, necessary to further explore the impressive capacity of locally accumulated metabolites to influence a wide range of health benefits. This could be realised by allocating study participants to groups following either a traditional strength training protocol or LMS-inducing training protocol, whilst also factoring in the perceived effort with its possible negative effect on exercise performance in the subsequent days. This could be prevented by separating upper and lower body muscle group sessions (Curovic et al., 2024), thus allowing a longer recovery until progressive adaptations are achieved.

## 5 Conclusion and future perspectives

Resistance exercise-induced LMS promotes conditions for strength and hypertrophy improvements with light loads by enabling mechanical tension on type 2 fibres in the anaerobic intramuscular environment. The LMS-aligned protocols lead to improved muscular endurance and reductions in blood pressure, while also demonstrating a notable potential for the improvement of metabolic and cognitive health. The most efficient strategy for inducing local metabolic response appears to involve targeted resistance training for a selected body region using light-to-moderate loads with the close proximity to failure achieved by the contraction duration or repetition number and/or shortened rest periods. Additionally, BFR resistance training could serve as a potent alternative for the mentioned physiological responses due to accelerated metabolite accumulation (i.e., lactate) in muscles after the onset of contractions. Furthermore, the regional metabolic stress obtained by high-volume exercise of one body segment (e.g., lower body) could facilitate adaptations from isolate strength-trained muscles in remote body segments (e.g., upper extremities) via stimulated neurophysiological mechanisms (Curovic et al., 2024). Therefore, it may be the time to shift the focus from comparing “advanced training techniques” to traditional routines solely for (local) strength and hypertrophy improvements (Weakley et al., 2023; Curovic et al., 2024; Grønfeldt et al., 2020), and instead start evaluating their impact on a broader range of outcomes (Hjortshoej et al., 2023; Angelopoulos et al., 2023; Miller et al., 2021). Randomised controlled trials comparing LMS protocols with traditional strength training interventions could provide valuable insights into the magnitude of those effects via incorporating wide range of functional assessments (e.g., strength qualities, muscular endurance, aerobic power, cognitive processing tests) and biochemical circulating factors (e.g., lactates, PGC – 1α group, BDNF, irisin, decorin, folistatin, myostatin, interleukins) with their influence toward cardiometabolic adaptations (e.g., insulin sensitivity, lipid profile, blood pressure).
